# Taking good retinal images for DR screening: considerations and practical tips

**Published:** 2023-07-07

**Authors:** Sharon Tench-Norbal

**Affiliations:** 1 Family Nurse Practitioner and DR Focal Point-Screener/Grader: Ministry of Health, Wellness and Elderly Affairs, Castries, St Lucia.

**Taking good retinal images is a vital step in screening for diabetic retinopathy. All measures should be taken to ensure that the images captured are relevant and of sufficiently good quality to ensure that no-one is misdiagnosed.**

**Figure:**
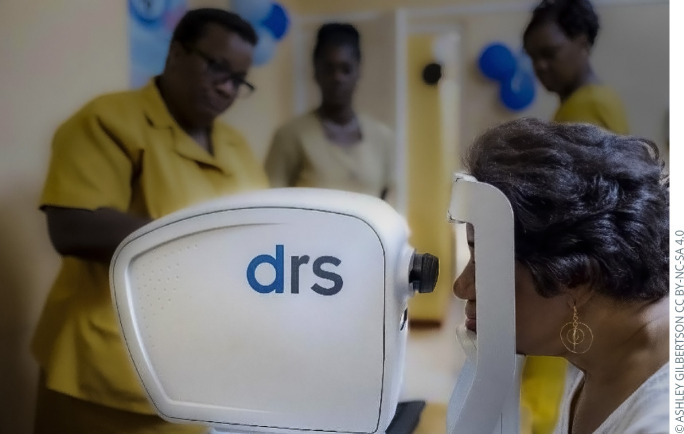
Diabetic retinopathy screening using a table-top retinal camera. The patient’s head is still and in the correct position. **ST LUCIA**

Diabetic eye screening allows people living with diabetes to benefit from early detection and treatment of sight-threatening diabetic retinopathy (DR). Screening is usually done by taking a photo of the back of the eye using a retinal camera (see article on page 4). These photos or images should be of good quality, so that the person grading them can make an accurate assessment and refer people who need treatment.

## What is a good quality image?

A good quality image is sufficiently clear to identify the anatomy of the fundus, including the macula, the optic disc, and the blood vessels. Commonly, photographs are taken in each eye: one of the optic disc (nasal image) and one of the macula (central image). These images are usually enough to enable the grader to identify any abnormalities. However, each image must be clear and in focus. If not, you will have to take extra images, which can be time consuming and lead to delays.

## Tips for capturing a good quality image

### 1. Prepare the patient

It is important to prepare the person by talking to them about what will happen, and why; this will help them to cooperate with you more fully. For example:

- Explain the reason for taking more than one photo of each eye and why they will be given dilation drops (if used).
- If using a camera with a flash, tell the person to expect a flash of light when the image is captured.
- If there is a target for them to look at, explain clearly what you want them to do, and why.

### 2. Consider pupil size

If a pupil is too small, the resulting images can be too dark and blurred to grade. For this reason many programmes include pupil dilation (by instilling drops of a mydriatic agent) in their DR screening guidelines. Recent technology advances include cameras that are able to take photographs without dilating the pupils, and these can be useful in field screening programmes where pupil dilation may not be practical.

If the person has difficulty focusing on a fixed point (for example, if they have poor vision or loss of vision in one eye, it may be useful to use an eye patch or eye occluder to cover the affected eye.

### 3. Position the patient correctly

When using a static camera, the person’s position at the camera can affect the quality of the image. Ensure that they are seated in a comfortable position. The use of a height-adjustable chair or table will help you to position the person’s chin and forehead correctly. Tell them to blink and then keep the eyes wide open until the image is taken. It may be necessary to hold up the eyelid; for example, in people with droopy eyelids. When using a hand-held camera, ensure that both the person and the photographer are in a comfortable position.

### 4. Keep the lens clean

Keep the camera lens free from anything that may obscure the image, such as eyelashes, dust, saliva droplets from speaking over the lens, or fingerprints – these cause some of the more common artefacts seen in retinal images, making them difficult to grade. Carefully clean the lens according to the manufacturer’s instructions, taking care not to scratch it.

### 5. Avoid bright light

A room which is brightly lit may cause the image to appear over-exposed at the centre, and darker or opaque at the outer edges. It is therefore better to take the photographs in a darkened room.

## Challenges

There are situations where, despite doing your best and taking all precautions, the images are still not good enough to confidently grade the level of diabetic retinopathy. One example is when patients have cataract or corneal opacities that obscure the view of the retina. These patients should be referred to the eye clinic and the images classified as ungradable. Ensure that these patients are followed up and/or are referred back for DR screening after surgery.

